# 以肺纤维化就诊的原发性纵膈精原细胞瘤1例

**DOI:** 10.3779/j.issn.1009-3419.2013.10.12

**Published:** 2013-10-20

**Authors:** 虹 张, 颖 杜, 伟 刘, 葆琳 魏, 增涛 孙, 建功 胡

**Affiliations:** 1 300150 天津，天津中医药大学第二附属医院呼吸科 Department of Respiratory Medicine, the Second Affiliated Hospital of Tianjin University of Traditional Chinese Medicine, Tianjin 300150, China; 2 300150 天津，天津中医药大学第二附属医院病理科 Department of Pathology, the Second Affiliated Hospital of Tianjin University of Traditional Chinese Medicine, Tianjin 300150, China

## 1 一般资料

患者王某，男性，65岁，主因“间断喘憋1年，加重1月”于2013年5月2日就诊于我院。既往高血压、慢性消化道溃疡病史。患者入院1年前无明显诱因出现咳嗽咯痰、喘憋，于2012年7月30日首次就诊于我院呼吸科，查胸计算机断层扫描（computed tomography, CT）示“两肺间质纤维化，肺气肿，两下肺炎症，两侧包裹性积液”，肺功能示“中重度限制性通气功能障碍，弥散量中度降低”，肿瘤标记物未见异常，予抗炎化痰平喘治疗后好转出院。2012年11月26日患者主因“上腹隐痛3月余，加重1周”于我院消化科住院治疗，住院期间查胃镜示“慢性胃炎”，胸CT示“两肺间质纤维化伴炎症，两侧胸膜增厚，右侧包裹性胸腔积液”，肿瘤标记物无异常，予对症治疗后好转出院。此后患者咳喘时有发作、纳差，近1年来体重下降20余斤。

此次入院前一月患者喘憋持续加重，外院查胸片示“两肺间质纤维化、右侧胸腔积液”，遂以“肺纤维化、胸腔积液”收入我院呼吸科住院治疗。入院查体：T 36.6 ℃，BP 130/80 mmHg，神清，口唇紫绀，双锁骨上窝淋巴结肿大，颈后可见一大小约0.8 cm×1.0 cm肿大淋巴结，压痛明显，双肺呼吸音低，可闻及细小爆裂音，HR 86次/分，心律不齐，腹（-），双下肢无水肿。予超声胸水探查定位后行胸腔细管引流术，引出橘黄色透明胸腔积液并送检。结合患者胸水常规、生化，考虑为渗出液，胸水癌胚抗原（carcino-embryonic antigen, CEA）6.40 ng/mL，然胸水多次送病理检测均未找到肿瘤细胞，仅可见少量淋巴细胞、中性粒细胞。胸腔积液引流后查胸CT示“①两肺间质纤维化伴炎症；②肺气肿、肺大泡；③纵膈密度增高，其内组织分界欠清并伴多发淋巴结增大”（图 1），肺功能示“重度限制性通气功能障碍，弥散量极重度降低”，肿瘤标记物示“CEA 23.1 ng/mL，细胞角蛋白21-1（cytokeratins 21-1, CYFRA21-1）4.92 ng/mL，糖蛋白抗原199（carbohydrate antigen199, CA199），822.37 U/mL，鳞状上皮细胞癌抗原（squamous cell carcinoma antigen, SCC）1.88 ng/mL”，均明显升高。遂予超声气管镜引导下行第7组纵膈淋巴结穿刺活检，气管镜下示“隆突增宽，粘膜轻度充血”，纵膈穿刺刷片找到高度可疑肿瘤细胞，隆突下淋巴结穿刺物病理：送检灰白灰粉不整形组织一堆，直径0.7 cm：（隆突下淋巴结穿刺物）为少量退变的纤维及坏死组织，内散在异形细胞；免疫组化：白细胞分化抗原20（cluster of differentiation 20, CD20）、白细胞分化抗原3（CD3）（-）、细胞角蛋白7（cytokeratin 7, CK7）（+）、甲状腺转录因子-1（thyroid transcription factor-1, TTF-1）（+）、细胞角蛋白34βE12（cytokeratin 34βE12, CK34βE12）（+/-）、P63（-），倾向为腺癌（图 2A，图 2B）。为明确纵膈肿瘤细胞来源，予局麻下行左颈后淋巴结摘除活检术，病理示：送检灰粉结节样肿物一个，大小1.5 cm×1.1 cm×0.7 cm，切面灰白、灰红、质软；（颈左侧）淋巴结结构破坏，淋巴结结构破坏，内可见较大的肿瘤细胞，实性、巢状、腺样排列，考虑为精原细胞瘤。免疫组化：胎盘碱性磷酸酶（placental alkaline phosphatase, PLAP）（+）、肌酸激酶（creatine kinase, CK）（+）、波形蛋白（vimentin）（+/-）、白细胞分化抗原117（CD117）（+），病变符合精原细胞瘤（图 2C，图 2D）。后患者家属将颈后淋巴结病理涂片送至天津医科大学肿瘤医院，亦证实其为“淋巴结转移性低分化恶性肿瘤，结合免疫组化结果，考虑为生殖细胞来源肿瘤（精原细胞瘤）”。患者无会阴部不适主诉，彩超示“双侧睾丸大小正常，内部回声均匀，彩色多普勒超声（color Doppler flow imaging，CDFI）显示未见异常血流信号”。至此，患者明确诊断为“原发性纵膈精原细胞瘤”。其胸CT显示肺间质呈网状结节影、支气管束增粗、小叶间隔呈串珠形增厚、两侧胸膜增厚等均考虑为“癌性淋巴管炎”。系因肿瘤细胞在淋巴管内生长繁殖，或/及淋巴引流受阻，都使淋巴管扩张，局部可出现间质性肺水肿，致使间质性病变加重，多见于淋巴管及结缔组织丰富的支气管血管周围，小叶间隔及胸膜下区域。诊断明确后予顺铂+依托泊苷化疗1周期。经治疗，患者喘憋症状好转出院。

**1:**
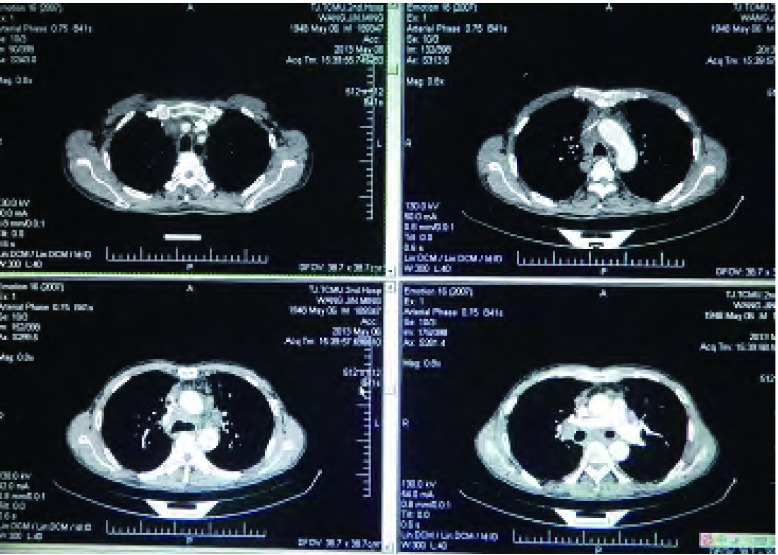
胸部CT示：纵膈密度增高，其内组织分界欠清并伴多发淋巴结增大 The chest CT showed: mediastinal density increased, obscure boundary with multiple enlarged lymph nodes. CT: computed tomography.

**2:**
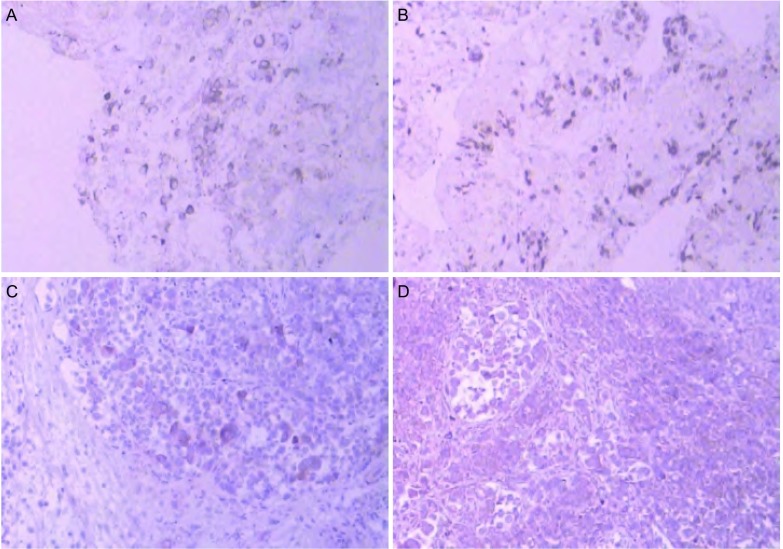
免疫组化图片（iVision, ×100）。A：细胞角蛋白7（cytokeratin7, CK7）（+）；B：甲状腺转录因子-1（thyroid transcription factor-1, TTF-1）（+）；C：胎盘碱性磷酸酶（placental alkaline phosphatase, PLAP）（+）；D：白细胞分化抗原117（cluster of differentiation 117, CD117）（+）。 Immunohistochemical images (iVision, ×100). A: CK7 (+); B: TTF-1 (+); C: PLAP (+); D: CD117 (+).

## 2 讨论

精原细胞瘤（seminoma）也叫生殖细胞癌（germino carcinoma），起源胚胎发育过程中残留于生殖细胞的恶性肿瘤^[1]^，是睾丸最常见的肿瘤。恶性生殖细胞肿瘤通常发生于性腺，但也有部分病例发生于性腺外，如纵膈、腹膜后腔、松果体^[2]^。刘仁伟等^[3]^在对7例精原细胞瘤患者的影像学表现进行回顾性分析的过程中发现：纵膈原发性精原细胞瘤的CT表现为前中上纵膈内大的不规则软组织肿块，肿瘤浸润生长，大血管周围脂肪间隙消失，肿瘤内部及边缘无钙化，肿瘤内部可有点片状坏死灶，可伴有上腔静脉综合征，可存在淋巴结转移或胸膜种植转移。最终明确诊断仍需依靠病理及免疫组化。原发纵膈精原细胞瘤生长缓慢、发生部位隐匿，症状多出现在病程晚期，临床表现为肿块压迫产生的局限症状，如胸闷、胸痛、咳嗽、呼吸困难等，全身症状少见。部分严重者可伴发热、体重下降及上腔静脉阻塞综合征。原发性纵膈精原细胞瘤的临床表现、影像学特征与其他前纵膈肿瘤难以鉴别，其诊断需依靠手术活检或穿刺活检、胸腔积液找脱落细胞。其组织类型与睾丸精原细胞瘤相同。

在治疗方面，Bokemeyer等^[4]^报告的回顾性分析中，欧美11个中心于1975年-1996年以顺铂为基础的联合化疗治疗原发性纵膈精原细胞瘤，其治愈率近90%。原发性纵膈精原细胞瘤为中低度恶性肿瘤，对放疗、化疗敏感，为放疗、化疗可治愈的肿瘤之一，预后较好。5年生存率为50%-80%，10年生存率为65% -69%^[5]^。Hurt^[6]^认为有以下情况者预后较差：①年龄 > 35岁；②伴发热；③伴上腔静脉阻塞综合征；④锁骨上窝或颈静脉淋巴结肿大；⑤胸片检查示肺门病灶。该病发病隐匿，患者就诊时多已失去手术时机，手术完全切除率仅为20%^[7]^。

本例患者1年内因喘憋、纳差三次住院治疗，前两次住院期间查胸CT均示双肺间质纤维化伴炎症，无纵膈淋巴结肿大等异常影像。然针对肺纤维化对症治疗后喘憋无明显改善。此次住院期间查胸CT才显示出纵膈肿物，伴多发淋巴结肿大，后经进一步检查，得以明确诊断“原发性纵膈精原细胞瘤、伴多发淋巴结转移，肺部癌性淋巴管炎”。说明原发性纵膈精原细胞瘤发病隐匿，症状出现时通常已至病程晚期。因此在临床上对常见病常规治疗后症状无明显改善时，应提高警惕，进一步追查是否为疑难少见病，以明确诊断，防止漏诊误诊。
